# Producing Cochrane systematic reviews—a qualitative study of current approaches and opportunities for innovation and improvement

**DOI:** 10.1186/s13643-017-0542-3

**Published:** 2017-08-01

**Authors:** Tari Turner, Sally Green, David Tovey, Steve McDonald, Karla Soares-Weiser, Charlotte Pestridge, Julian Elliott, on behalf of the Project Transform Team, Julian Elliott, James Thomas, Sally Green, Chris Mavergames, Steve McDonald, Anna Noel-Storr, David Tovey, Tari Turner, Mike Clarke, Paul Glasziou, Clive Adams, Lorne Becker, Linn Brandt, Rachel Churchill, Agustin Ciapponi, Gordon Dooley, Ruth Foxlee, Demian Glujovsky, Toby Lasserson, Geraldine Macdonald, Sue Marcus, Rupert McShane, Melissa Murano, Charlotte Pestridge, Daniel Perez Rada, Gabriel Rada, Jacob Riis, Ian Shemilt, Emily Steele, Anneliese Synnot, Chris Watts, Karla Soares-Weiser, IKMD developers

**Affiliations:** 1 Cochrane Australia, School of Public Health and Preventive Medicine, Monash University, Melbourne, Australia; 2 Cochrane Editorial Unit, Cochrane, London, UK; 3 Cochrane Innovations, Cochrane, London, UK

**Keywords:** Systematic review, Methods, Quality, Innovation, Technology, Editorial production processes

## Abstract

**Background:**

Producing high-quality, relevant systematic reviews and keeping them up to date is challenging. Cochrane is a leading provider of systematic reviews in health. For Cochrane to continue to contribute to improvements in heath, Cochrane Reviews must be rigorous, reliable and up to date. We aimed to explore existing models of Cochrane Review production and emerging opportunities to improve the efficiency and sustainability of these processes.

**Methods:**

To inform discussions about how to best achieve this, we conducted 26 interviews and an online survey with 106 respondents.

**Results:**

Respondents highlighted the importance and challenge of creating reliable, timely systematic reviews. They described the challenges and opportunities presented by current production models, and they shared what they are doing to improve review production.

They particularly highlighted significant challenges with increasing complexity of review methods; difficulty keeping authors on board and on track; and the length of time required to complete the process. Strong themes emerged about the roles of authors and Review Groups, the central actors in the review production process.

The results suggest that improvements to Cochrane’s systematic review production models could come from improving clarity of roles and expectations, ensuring continuity and consistency of input, enabling active management of the review process, centralising some review production steps; breaking reviews into smaller “chunks”, and improving approaches to building capacity of and sharing information between authors and Review Groups.

Respondents noted the important role new technologies have to play in enabling these improvements.

**Conclusions:**

The findings of this study will inform the development of new Cochrane Review production models and may provide valuable data for other systematic review producers as they consider how best to produce rigorous, reliable, up-to-date reviews.

## Background

Systematic reviews gather and synthesise the best evidence from research to support informed healthcare decisions by patients, health professionals and policy-makers. As such, systematic reviews are a vital step in the translation of the results of research into practice and policy to improve healthcare outcomes [1, 2].

Producing high-quality, relevant systematic reviews and keeping them up to date are challenging [3, 4]. The increasing methodological complexity of the review process and the rapidly expanding body of evidence available for review add to the difficulty—and the impact of both factors is only likely to increase in the future [3].

Cochrane is a leading provider of systematic reviews in health. Cochrane Reviews are prepared by review author teams, working with Cochrane Review Groups. Each Cochrane Review Group is responsible for a specific area of health care or policy. The Cochrane Database of Systematic Reviews now includes more than 6500 systematic reviews, with another 2500 reviews currently underway [5].

Cochrane has been producing systematic reviews for more than 20 years. In recognition of the changing environment and potential role of new technologies, Cochrane is undertaking a range of initiatives to identify and scale up new ways of working that ensure Cochrane Reviews continue to be relevant and reliable and that review production is efficient and sustainable.

While a number of studies have been conducted exploring the quality and impact of Cochrane Reviews [6], we are not aware of any published research documenting how reviews are produced within Cochrane and how this could be improved.

This study explores systematic review production models currently employed within and beyond Cochrane. It highlights what people are doing to improve the quality and timeliness of Cochrane systematic review production; what is working, what challenges they are facing and what is needed to enable further improvement.

## Methods

We aimed to understand the strengths and weaknesses of the processes by which systematic reviews are currently being produced within and beyond Cochrane and to identify opportunities for improvement to enable efficient and sustainable production of reliable, relevant, up-to-date Cochrane Reviews.

We used a primarily qualitative approach with some embedded quantitative methods to explore participants’ experiences with systematic review production models, including both an online survey and semi-structured interviews.

Ethics approval was provided by Monash University (CF15/2995 – 2015001229).

### Participants

#### Interviews

Potential interview participants were from two groups:

1. Participants associated with Cochrane Review production

This group included the following:

- Cochrane Review authors
- Editors and staff of Cochrane Review Groups (the 52 Review Groups responsible for producing Cochrane reviews in health-related domains)
- Members of the Cochrane Editorial Unit (the central Cochrane unit with responsibility for supporting Cochrane groups to improve the quality of Cochrane Reviews)
- Members of Cochrane Methods Groups (the 17 Methods Groups responsible for developing and advising on methods for Cochrane Reviews);
- Staff of Cochrane Centres (Cochrane’s geographical representatives in more than 43 countries and regions)
- Members of the Cochrane Central Executive Team

2. Participants associated with non-Cochrane systematic review production

This group included professionals working in systematic review production for systematic review firms, health technology assessment and clinical practice guideline development groups.

Potential participants were invited to participate in an interview through existing communication channels for Cochrane including the Cochrane website, newsletters, email lists, social media and other similar methods. Individuals known to the investigator team were directly emailed invitations to participate in the interview and encouraged to forward the invitation to other potential participants.

Participation was voluntary. Potential participants were provided with a participant information statement by email prior to the interview and provided evidence of informed consent by return email.

#### Surveys

Potential survey participants were associated with Cochrane Review production and included the following:

- Cochrane Review authors
- Editors and staff of Cochrane Review Groups
- Members of the Cochrane Editorial Unit
- Members of Cochrane Methods Groups
- Staff of Cochrane Centres
- Members of the Cochrane Central Executive Team

We planned to recruit 30–50 survey respondents.

Participants were invited to complete the survey through existing communication channels for Cochrane including the Cochrane website, newsletters, email lists and social media. Individuals identified by the investigator team were also directly emailed invitations to participate in the survey and encouraged to forward the invitation to other potential participants.

Participation was voluntary. Potential participants were invited to visit the survey webpage which provided a participant information statement. Choosing to proceed with the survey constituted informed consent.

### Data collection

#### Interviews

Data were collected using a semi-structured interview in person or by phone, Skype or similar technology. The interview schedule was developed by TT in consultation with the other authors and pilot-tested before use.

Interview questions were loosely based on a pre-determined interview schedule, with questions varied so as to be relevant to the interviewee’s role and experience and in the light of responses to preceding questions. Detailed notes were taken during the interviews. Interview questions explored the interviewee’s experience of different approaches to review production and their reflections on the benefits and weaknesses of these different approaches. Interviewees were asked to describe which characteristics of review production processes contribute most to high-quality, timely review production; what the most common challenges are; and where there are opportunities to improve the current production model.

Interviews were conducted by TT, a Cochrane Review author and systematic review methodologist who is familiar with but has no authoritative role within Cochrane. TT is an experienced qualitative interviewer.

#### Survey

Data were collected using an online survey tool. The survey included demographic questions, 18 close-ended and 10 open-ended questions. The close-ended questions asked respondents to rate a set of review production characteristics in terms of their importance to review quality, importance to review timeliness and difficulty to achieve.

The open-ended questions sought information on respondents’ experiences of how the review production processes to which they had contributed had been organised. Respondents were asked to reflect on times that these processes had worked well, leading to production of high-quality, useful, timely reviews, and describe how the review production was organised. Similarly, respondents were asked to consider when these processes had not worked well and identify elements of the process that may have contributed to the poorer outcomes. Respondents were prompted to consider characteristics such as team formation, team management, roles within teams, managing quality and time expectations, author team skill mix, structure of and support for review teams and accountability mechanisms and reflect on what approaches had been most effective in their experience. Respondents were also invited to suggest opportunities to improve the current review production model.

Participants could choose whether to provide their contact details to enable participation in a follow-up interview.

### Data analysis

NVivo 10 (http://www.qsrinternational.com/nvivo-support/downloads/nvivo10-for-windows) was used to analyse qualitative data and to extract quotes. Thematic analysis was undertaken of interview notes and qualitative survey responses. These were initially analysed using open coding to identify key concepts which were then collapsed into emerging themes. TT undertook the primary data analysis. SG and JE reviewed a subset of the coding and collaborated on the conceptual development of themes. A draft report of the analysis was provided to interview respondents and survey respondents who provided contact information, for feedback.

Qualitative data from interviews were initially analysed independently of the data from the open-ended questions in the survey; however, as the similarity of themes became apparent, the analysis was combined.

## Results

### Respondents

We conducted 26 interviews between July and November 2015, until data saturation was reached. Interview participants included Cochrane Managing Editors of Cochrane Review Groups (6), authors of Cochrane Reviews (6), Review Group staff (other than Editors) (3), Coordinating Editors of Cochrane Review Groups (2), Editors (other than Managing or Coordinating Editors) (2), Cochrane Consumer contributors (1) and systematic review authors and guideline developers from outside Cochrane (6). The interview participants were from seven countries and included six participants with a first language other than English and three participants from low- and middle-income countries.

One hundred six people provided online survey responses between August and October 2015. Survey participants described themselves as Authors (47), Managing or Coordinating Editors (29), Information Specialists (13), Consumers (15), Methodologists (11), Editors (5), Centre Staff (2), CEU staff (1), and Other (8) (multiple responses allowed). The respondents had a median of 10 years of Cochrane experience (range 0.4–20). The survey respondents provided extensive answers to the open-ended questions, with 52 respondents (49%) providing at least one answer to an open-ended question and a total of more than 14,000 words of free-text responses.

External respondents were very familiar with Cochrane Review production and had useful insights about aspects of their own review production approaches that might translate well into the Cochrane context or could catalyse new ways of producing reviews within Cochrane. Except where specifically noted below, their responses were very strongly aligned with those of the respondents from within Cochrane.

### How are Cochrane Reviews produced?

The respondents described the importance and the challenge of creating high-quality, relevant, up-to-date Cochrane systematic reviews. Their responses highlighted that while the methods of Cochrane Review production are very systematic, the production process is also variable and creative. It was also clear that there is substantial variation in the approaches taken to review production both between, and also within, Cochrane Review Groups. Almost every aspect of the review production process varies to some extent between groups.

Similarly, the respondents noted that while each individual review ostensibly follows the same approach, they are not predictable. Reviews are undertaken in an environment that is volatile and frequently under-resourced, and they are undertaken by people with multiple competing commitments and widely varying levels of skill and availability. Review topics cover the full gamut from incredibly narrow to courageously broad, and vary, often unforeseeably, in complexity. Each review presents its own unexpected challenges.

However, across the respondents strong themes emerged about the roles of authors and Review Groups, the central actors in the review production process.

#### Working effectively with, and within, author teams is critical and challenging

Cochrane Review authors are at the centre of review production. The respondents frequently emphasised that good relationships between the Cochrane Review Group and the author team, and within author teams, were crucial to good review production processes.

that’s the ‘collaboration’ bit of The Cochrane Collaboration... it’s all about the human relationships (Managing Editor)

good people drive good processes (Author, Editor)

##### Team formation

There was a shared sense that while the make-up of the author team is the key to successful review production, in most circumstances, processes for formation of author teams and task allocation within author teams are informal and organic. This was not perceived to be problematic.

Team formation is organic, ask around, bring people together, there is no process (Coordinating Editor, Author)

[We] have tried a prescriptive process, and wouldn’t recommend it (Managing Editor)

##### Author requirements

Requirements for author teams vary, with some Review Groups having very stringent requirements and others determined to accept all comers. However, the respondents agreed on the importance of having an effective review team leader. There was also acknowledgement that while requirements for authors (e.g. having a team member who has previously conducted a Cochrane Review) are often useful, they are not enough to ensure success.

Some people just get on and do it, and you can’t predict who will (Managing Editor)

Linked to this, there was also a clear tension (both within and between Review Groups) between focusing on capacity building (with benefits being bringing in new authors and the greater time availability of junior team members) and the benefits of experienced authors who require less hands-on support. Review Groups have strongly held widely varying positions on this.

Abandon inclusiveness regarding new authors (Author, Editor, Methodologist)

[Our] group will take anybody who wants to do a review, work with the enthusiasm of people, … let them do it as long as they do it well (Author, Coordinating Editor)

Many respondents highlighted the importance and difficulty of sourcing timely clinical input throughout the review process and of not wasting this precious resource on tedious tasks like screening search returns.

#### Payment and incentives

The respondents described a wide range of payment and incentive models for author teams. These included fully paid teams, outsourcing to private companies, teams with some paid members (often Review Group or a partner organisation’s staff), stipends for travel to the Review Group office or similar and fully volunteer teams. All of these approaches were perceived to have strengths and weaknesses.

##### Paid authors

Having been funded, protected research time for the lead author was a frequent suggestion for improving the timeliness of review production.

Have allocated funding to do the review really helps – especially for dedicated time of the lead author (Deputy Coordinating Editor, Author)

##### At partner organisations

Funding staff at partner organisations to conduct reviews was seen as a good way to build capacity, particularly when partner organisations were in low- and middle-income countries (LMICs). However, the respondents also highlighted weaknesses with this model, with paid authors likely to end up being responsible for many reviews, potentially across several Review Groups, and difficulties arising with accountability.

[A downside] is one individual ends up being responsible for a lot of reviews. If employed for several years and do 2 to 4 reviews per year, after 5 years they will have 20 reviews to manage, and these sit across Review Groups, so pressure not just from [our Review Group], but also from other Review Groups. (Managing Editor)

I’m not their line manager, and neither is my line manager, [this is a] con in terms of how we work with partners. (Managing Editor)

##### At Review Groups

Employing staff at Review Groups to lead or support reviews was frequently suggested as an effective model, although respondents questioned the feasibility of employing fulltime staff in lower resource settings.

I am a firm believer that Review Groups need to employ researchers to lead reviews, particularly to do the donkey work, like data extraction (Consumer, Author, Editor)

Our best experiences have tended to come with motivated author teams who have some dedicated time to carry out the review and have an experienced author on the team. This works particularly well when that person is employed at the editorial base and can facilitate good communication between the authors and the editorial base. This has tended to produce the most timely, high quality reviews. (Author, Consumer, Editor)

In developed world, the model of commissioning, fulltime researchers; it’s not suitable for LMICs (Author)

##### Volunteers

The respondents were quick to point out that while funded or partially funded teams were often more productive, there were both philosophical and practical reasons why volunteers should remain at the heart of Cochrane.

[I would] Hate to see Cochrane only having paid staff, but as part of the team it is essential (Author, Editor)

The respondents highlighted the key role of volunteer Cochrane authors, both in producing the bulk of Cochrane Reviews and in producing a wide variety of reviews and also in maintaining the connection between clinicians and community members and the research that was of interest to them.

Working with, and as, volunteer authors was acknowledged to be very challenging, but incredibly valuable.

With volunteers it is very difficult, but volunteers are the only way Cochrane can be as productive as it is (Consumer, Author, Editor)

The respondents highlighted the increasing difficulty of finding volunteers with available, flexible time to work on Cochrane Reviews, both in LMICs and in high-resource settings as the funding model changes.

It’s hard to see how a completely voluntary model is sustainable at scale in those [LMIC] settings. (Author, Coordinating Editor)

##### Commercial systematic review producers

A small number of Review Groups described their experiences of contracting commercial systematic review producers for some or all of the review process. While obviously dependent on availability of funding, these arrangements were seen to be very efficient at producing high-quality reviews quickly.

Companies are far more efficient than universities, lower overheads, more nimble (Coordinating Editor, Author)

##### Incentives and fellowships

Small incentives and fellowships were felt to be useful in providing legitimacy for review work for both Review Groups and authors, facilitating project management by Review Groups and allowing authors to demonstrate the value of review work to their institutions. Fellowships for authors to visit Review Groups were particularly valued for the opportunity they provided to work face to face.

… we have had some success with monetary incentives … that come with non-negotiable publication deadlines. This allows the editorial base to legitimately … set deadlines with the review team, as well as providing the opportunity of offering peer referees a small incentive in return for prompt feedback. (Managing Editor)

A previous round of work with that same Cochrane group had been given minimal funding for some updates ... This in no way covered the costs of the work, but it helped to oil the wheels in some universities and allowed one of us to attend a Cochrane symposium. (Author)

Ability to work face-to-face is invaluable (Managing Editor)

There was widespread acceptance that having some funding makes author teams more likely to successfully complete their review, largely by leading to a greater sense of accountability. However, this was paralleled by acknowledgement that funding does not solve all review production problems.

Things move along in a different way where there is a little funding for the review team to justify spending the time (Managing Editor)

### The role of Review Groups varies widely and can be unclear

#### Project management

Project management of review production processes emerged as a substantive issue. The extent of project management undertaken by Review Groups varies widely with some groups providing active, hands-on management by one of the following: the Managing Editor, a paid lead reviewer (often a member of the Review Group staff) or unpaid guarantor authors. Usually, Review Groups provide this level of active project management for a small selection of priority reviews; however, some aim to cover all reviews. Other Review Groups describe themselves as “hands-off” and do not provide any project management of review production. There is a sense of an overall trend towards more hands-on management, although this is acknowledged to be very resource intensive and sometimes perceived to be unattainable.

If you can find a Review Group where people are less hands-on than we are and are producing good reviews, I’d like to see it (Coordinating Editor, Author)

#### The role of Review Groups

There is also considerable variation in how Review Groups perceive their wider role in the review production process. This leads to substantial differences in the extent to which Review Groups see their role as providing clinical input, methodological guidance, editorial oversight and/or author support.

#### Conflation of review publication and review production

A related theme was the conflation of review production and review publishing roles. The respondents were very concerned about the challenges this conflation creates in the production process (see, for example, discussion of peer review below). They also highlighted that this leads to, at the very least, a perceived conflict of interest for Review Groups, which are responsible for both producing the review and making the decision about when a review can be published. There was a perception of a potential for there to be perverse incentives for Review Groups to publish reviews that were not of the highest possible quality.

[The] production process is currently confused with publication process (Author)

#### Peer review

Substantial issues with peer review processes were mentioned by many respondents, both authors and individuals within Review Groups. These issues include the long time delays introduced by peer review processes; lack of clarity of the purpose of peer review; difficulty sourcing appropriate, high-quality peer review; and challenges with collating and communicating the feedback in a constructive, helpful way for authors.

Best peer review is from editors. External peer review is cosmetic. (Coordinating Editor, Author)

Peer refereeing process timeframes are terrible. Can be months before feedback is received, then authors respond, then another round of feedback. Can be 6-12 months, which is very demotivating. (Author, Editor)

### What is needed in a new approach?

Most respondents from within Cochrane were either actively looking for or actively trialling new approaches to review production.

The ongoing and ubiquitous search for new approaches highlights that there is shared understanding that solutions are urgently needed to the challenges that review production face and also that there is strong motivation to find effective ways to improve review production.

While the approaches to improvement being explored by the respondents differed, most seem designed to address a similar set of issues.

### Improved clarity of roles and expectations

Many of the challenges with current review production processes raised by the respondents appeared to be due to underlying lack of clarity about the roles and responsibilities of different contributors to the review process, particularly authors and Review Groups, and to a lesser extent Methods Groups.

The respondents described challenges arising from a lack of clarity of expectations about the following:

- Review quality, in terms of both methodological standards and standard of writing
- Timelines in terms of turnaround of review stages by both authors and Review Groups
- Roles of author teams and Review Groups in the review production process
  There is a big difference in expectations of authors. Some don’t communicate with the Review Group at all. Some think that we are going to write it for them. (Deputy Coordinating Editor, Author)
  A clear team leader is key. Also clarity of roles at outset. (Author, Managing Editor)

There was a feeling that one reason for poor review timeliness was a lack of clear understanding on the part of authors of the true investment of time and effort required to complete a review.

Ensuring commitment on the part of the author team to complete the review was an important goal for Review Groups, though it was not clear how this could be achieved. Some Review Groups raised both the appeal of and their hesitancy to implement formal, signed agreements with author teams that explicitly set out expectations, roles and responsibilities for authors. One of the reasons for hesitancy was a recognition of their own inability to feasibly respond within agreed timelines.

If we could guarantee timely feedback [as a Review Group] then maybe we could expect timely input from authors too (Author, Editor)

Several respondents emphasised the importance of getting off to a good start: the value of investing time to ensure clarity at the beginning of a review process in order to avoid later issues with the review. Approaches to improving clarity and commitment in the early stages of the review production process included increasing the rigour of title registration processes, extending protocol processes and raising author team requirements to ensure that reviews start well and avoid pitfalls later in the process.

The perception is that publishing the protocol is straightforward, it’s not, and it’s an important starting point for the review. Being proactive at this stage is helpful (Deputy Coordinating Editor, Author)

The start has it all. (Author)

[Our approach is] Massive frontloading of input, review proposals and protocols. [We are] Thinking about asking for Summary of Findings tables at protocol stage (Managing Editor, Author)

The respondents also noted that there was an expectation that reviews would use increasingly complex methods. They highlighted that this increased complexity led to increased workload and timelines for both authors and Review Groups and suggested that there should be a renewed focus on simple, reliable, useful reviews.

Go back to basics… Stop expecting complexity in all reviews (Author)

### Increased continuity and consistency of input

The respondents highlighted the vital importance of enabling continuity and consistency of input throughout the review process. They wanted consistent clinical, methodological and editorial input from all contributors to the review, including the author team, Review Group, methodologists and editors (including copyedit), throughout the review lifecycle, and they were frustrated when they received conflicting advice, which often led to delays or rework. This idea was linked to the importance of having an effective author team leader, discussed further below.

Most important is that [a] project manager is across the whole process so it has continuity (Author, External Review Producer)

#### Consistency throughout the review production and update process

Authors particularly valued consistency of input in order to ensure that earlier decisions are not revisited—for example, that methods signed off at the protocol stage are not queried at review submission. Linked to this, some respondents suggested that systems that would allow staged quality assurance sign off at appropriate points within review production, rather than one final quality assurance review, would be beneficial in terms of quality and timeliness of reviews and author motivation.

The respondents suggested that it might be useful to extend the idea of the author team beyond a single review and beyond a single version of a review, to encompass a community that was responsible for the ongoing life of a review as a way of ensuring ongoing consistency and continuity of input.

We need to reduce dependence on a single author team to see a review from outset through years of updates. Teams should become much more dynamic; if someone has to drop out of a task, then there should be someone else who can take their place. Reviews should be owned explicitly by groups rather than authors. There should be author membership communities formed around topics or CRGs. (Coordinating Editor, Author)

#### Consistency across reviews

The benefits of ensuring consistency of review structure and content between reviews (and ideally between Review Groups) were also emphasised by the respondents. Many different approaches to ensuring this consistency were described, such as standardised methods sections, use of review and protocol templates, exemplar protocols/reviews, and development of suites of reviews with shared background, PICO elements, and methods sections. In some Review Groups, these approaches are standard practice, and in others, they are quite new and still controversial. These approaches were all felt to improve both the quality and the timeliness of review production, while reducing author workload.

The quality of review improved if authors were given highly structured protocol and review templates. Also, interim editorial checks on risk of bias tables and SoF [Summary of Findings] tables improved quality. (Coordinating Editor, Author, Methodologist)

#### Consistency across Review Groups

The author respondents often suggested that standardising approaches to review production within and between Review Groups would be an immensely valuable step forward. Authors often struggled with the differing expectations and roles of different Review Groups in the production process.

#### Technology to support consistency

The respondents noted the value of technology in supporting consistency in a variety of ways including creating, storing and making available reusable review content; enabling linking between related reviews; providing methods of crosschecking content within reviews to ensure internal consistency; and providing an audit trail that captures decisions that are made during the review production process.

If reviewing process can be made easier [by software and standardisation], people can focus on the bits that need thinking (Author)

### Active, explicit facilitation and management of the review process

Many respondents noted that a major predictor of high-quality timely review production is coordination of the review process by a responsible, experienced person who is explicitly tasked with overall project management of the review production process and is appropriately positioned and skilled to overcome any process hurdles. As well as providing (or enabling access to) methodological and content leadership, a key role for this person is to provide a clear channel of communication with the Review Group.

Some respondents suggested that this role should be taken by a lead author; others felt it was better performed by a member of staff of the Review Group, often a research fellow or research associate.

The respondents also highlighted that author support tools that are designed to support review authoring and which allow coordination of the work of multiple independent contributors have additional, substantial project management benefits in enabling oversight of progress.

Most author teams do not have a senior person to oversee and manage – technology could provide this (Author, External Review Producer)

One reviewer needs to lead (or project manage) the entire review and consult regularly with others, set deadlines and deliverables. That person needs to be the conduit for the editorial team and manage review submissions and revisions. Communication with the review team is essential. (Author)

#### Centralisation of some review steps

The current model of review production within Cochrane devolves responsibility for much of the review production process to the 52 Review Groups which are each responsible for reviews in particular health-related topic areas. The potential for centralisation of several aspects of review production to improve quality and timeliness of reviews was mentioned by a number of the respondents.

#### Study identification

This included centralisation of elements of searching for studies and other Information Specialist activity. The respondents linked this both to the need for increased specificity of searches (which was seen to be vital for improved timeliness of individual reviews) and the importance of development of registers of clinical trials to support review production more broadly. Automation and machine learning was seen as a key component of achieving increased specificity of searches.

Trials register[s] and central search is invaluable to Review Groups and authors (Managing Editor)

[There are ] Huge opportunities in technology… Particularly automation of early phase of reviews (Coordinating Editor, Author, Methodologist)

#### Methods support

There was a strong desire for Cochrane to provide a central source of methods support. Statistical methods were mentioned most frequently by both authors and Review Groups, with Review Groups highlighting that they often felt unable to access the guidance they needed to confidently advise their authors. Several respondents suggested that some form of central statistical support would be very valuable. Other respondents noted that for methods that were common for most reviews but that were “developed and owned” outside of Cochrane (e.g. GRADE, www.gradeworkinggroup.org), there needed to be an internal, central source of guidance for how these tools should be applied to Cochrane Reviews.

[T]here is an opportunity for increased central oversight of key methods (Deputy Coordinating Editor, Author)

We don’t have anyone we can ask methodological questions of (Deputy Coordinating Editor, Author)

#### Peer review

There was also suggestion of the potential for (at least partial) centralisation of peer review processes. However, the respondents felt that for this to be effective, there needed to be clarity about the purpose of peer review—whether it is intended to address some or all of methodological, editorial or clinical issues—and for the continuity of input mentioned above to be ensured.

### Flexibility in breaking reviews into smaller, skill-specific “chunks”

A number of the respondents introduced the idea that it would be useful to break review production into bite-size, manageable, skill-specific tasks that could be taken on by appropriately skilled people.

Some Review Groups already do this by, for example, giving author teams the option of having some tasks (e.g. summary of findings tables) completed by Review Group-based specialists.

Chunking of review tasks was seen as a useful approach to explicitly ensure there was a good match between the skills and effort required for the task and the skills and time availability of the person completing the task.

Chunking was also seen as a useful response to the increasing complexity of methods and as a way of focusing the limited time of clinical authors on the areas of the review where their input was most valuable.

The respondents described the value of technology in enabling chunking by presenting a systematic review as a series of linked tasks, in holding and synthesising data from multiple contributors and in providing an audit trail for review decisions and processes.

Very important to move on from thinking that everybody has to do every part of the review (Author, External Review Producer)

Chunking of the review processes into smaller, discrete pieces of work was also felt to enable clearer communication and understanding of the effort likely to be required in each phase of the review process. As mentioned above, lack of understanding of effort required to complete a review was perceived to be a major reason for drop-out of authors. Chunking was also felt to build author motivation by giving authors a sense of progress and achievement throughout the review process, rather than only acknowledging this at the final submission.

Later in review process [I create] a plan for completion that chunks the tasks into pieces that people can understand … Try to give them a sense of the measurable tasks (Review Group Staff, Author)

Some respondents also linked chunking of review tasks to an increased ability to involve authors and others who have a first language other than English in review production.

Could come to a situation where people do a review, or tasks, in their own language and translate at the end (Author, External Review Producer)

### Improved approaches to building capacity and sharing information

#### Capacity building

The respondents noted that there was a need not only for upskilling of authors and editors, particularly in new review methods, but also in other aspects of the production process, including communication and peer review. Review Groups were seen to be under-resourced, or ill-equipped, to deliver the kind of training and support needed to upskill authors in these complex areas.

It is often left to editorial bases (and MEs in particular) to ensure that Cochrane developments (e.g. RevMan updates; RoB/SoF/GRADE) are implemented by review teams and incorporated into current and future reviews; it would be nice to think that support for us is considered in any future models. (Managing Editor)

#### Information sharing

Several respondents noted that there would be benefits from improving coordination and communication of Review Group activity focusing on improving review production. Highlighting this, a number of respondents from Review Groups noted that they were undertaking pilot activities designed to improve review production, and they were often unaware that other groups were undertaking similar initiatives.

[I am] not clear how Review Groups are sharing materials about review production management. [Our Review Group] has materials, many Review Groups have these, how can we better share these, so that we are not duplicating effort on both technical and management information? (Coordinating Editor, Author)

## Discussion and conclusions

Cochrane is a leader in production of systematic reviews of healthcare interventions. Ensuring that Cochrane Reviews continue to meet high standards of quality in an environment with increasingly complex, extended and varying methods are a substantial challenge. Diverse data sources and geographically dispersed author teams add to this challenge and bring important new opportunities to improve the reliability, timeliness and usefulness of Cochrane Reviews.

The participants in this study agreed that there are important, achievable opportunities to improve the production of Cochrane Reviews. These include opportunities to the following:

- Clarify roles and expectations of authors and Cochrane Review Groups
- Ensure continuity and consistency of input into reviews
- Actively coordinate the review process
- Centralise some aspects of review production
- Break reviews into smaller ‘chunks’
- Improve approaches to capacity building and information sharing around review production

This information is being used in discussion with the Cochrane community to identify and develop new review production models than can be explored and piloted. We are currently piloting an approach to produce living systematic reviews [7], supported by machine learning and citizen science approaches, and are exploring pilots focusing on supporting larger author teams and novel approaches to support participation in review production by authors in low-resource settings. Consideration of these approaches is happening against a backdrop of broader, substantive structural change within Cochrane, particularly for Cochrane Groups, arising from the structure and function review [8].

The study was limited to the data provided by the 26 interviewees and 106 survey respondents, and this of course only captures a small amount of the variety within an organisation as broad and varied as Cochrane. However, the extent to which shared themes emerged, and the diversity of roles, locations, experience and background of the respondents, gives us some confidence in the results. By its nature, the study had a relatively narrow focus on systematic review production within Cochrane; however, the results may also be useful to other systematic review producers and particularly those who are interested in exploring whether and how new technologies such as machine learning and online collaborative platforms can support efficient review production.

The findings highlight the ways in which these and other new technologies offer the potential to support and coordinate review production by broadening participation, breaking review activity into smaller skill-based chunks and allowing greater insight into and more active management of teams producing reviews.

The effectiveness and ramifications of these new technological tools, as well as the potential process changes, need to be further investigated, and their implications for authors, editors, evidence users and the review publication process need to be understood before their use can become widespread.

Well implemented, these opportunities could markedly improve the ability of systematic reviewers, such as Cochrane, to efficiently and sustainably produce high-quality, reliable systematic reviews that can support the translation of the results of research into practice and, ultimately, improve healthcare outcomes.

## Abbreviations

GRADE: Grading of Recommendations Assessment, Development and Evaluation
LMIC: Low- and middle-income countries
ME: Managing Editor
RG: Review Group
RoB: Risk of bias
SoF: Summary of findings
